# MinION nanopore sequencing identifies the position and structure of bacterial antibiotic resistance determinants in a multidrug-resistant strain of enteroaggregative *Escherichia coli*

**DOI:** 10.1099/mgen.0.000213

**Published:** 2018-09-20

**Authors:** David R. Greig, Timothy J. Dallman, Katie L. Hopkins, Claire Jenkins

**Affiliations:** Public Health England, UK

**Keywords:** nanopore sequencing, mobile genetic elements

## Abstract

The aim of this study was to use single-molecule, nanopore sequencing to explore the genomic environment of the resistance determinants in a multidrug-resistant (MDR) strain of enteroaggregative *Escherichia coli* serotype O51 : H30, sequence type (ST) 38. Sequencing was performed on the MinION Flow cell MIN-106 R9.4. Nanopore raw FAST5 reads were base-called using Albacore v1.2.1, converted to FASTA and FASTQ formats using Poretools v0.6.0, and assembled using Unicycler v0.4.2, combining the long-read sequencing data with short-read data produced by Illumina sequencing. The genome was interrogated against an antimicrobial resistance (AMR) gene reference database using blast. The majority of the 12 AMR determinants identified were clustered together on the chromosome at three separate locations flanked by integrases and/or insertion elements [region 1 –*catA*, *bla*_OXA-1_, *aac(6′)-Ib-*cr, *tetA* and *bla*_CTX-M-15_; region 2 – *dfrA1* and *aadA1*; region 3 – *catA*, *bla*_TEM-1_, *tetA* and *sul2*]. AMR determinants located outside these three regions were a chromosomally encoded *bla*_CMY-16_, mutations in *gyrA* and *parC*, and two plasmid-encoded AMR determinants, *bla*_OXA-181_ and *qnrS1* located on the same IncX3 plasmid. Long-read analysis of whole genome sequencing data identified mobile genetic elements on which AMR determinants were located and revealed the combination of different AMR determinants co-located on the same mobile element. These data contribute to a better understanding of the transmission of co-located AMR determinants in MDR *E. coli* causing gastrointestinal and extra-intestinal infections.

## Data Summary

Long- and short-read FASTQ sequences have been deposited in the NCBI Sequence Read Archive under the BioProject PRJNA315192 (SRP071789), Sample: SRS2937479, Experiment: SRX3676443, Accession numbers Nanopore FASTQ: SRR6702264 and Illumina FASTQ: SRR5470155. Assembly accession numbers: CP026723.1 (chromosome) and CP026724.1 to CP026728.1 (five individual plasmids).

**Impact Statement**

The implementation of whole genome sequencing (WGS) for routine public health surveillance has enabled us to monitor trends in antimicrobial resistance (AMR) gene content in *Escherichia coli* and provides real-time data on emerging resistance patterns nationally and internationally. Understanding the genomic environment of these AMR determinants, with respect to whether they are chromosomally encoded or plasmid-encoded, and whether they are co-located is essential for monitoring transmission of AMR and assessing the risk to public health. Short-read WGS data can identify the presence or absence of AMR determinants but not their genomic architecture. In this study, long-read analysis of WGS data enabled us to determine the mobile genetic elements on which the key AMR determinants are located, and to characterize the combination of different AMR determinants co-located on the same mobile element. A combination of short- and long-read WGS data contributes to a better understanding of the transmission of co-located AMR determinants in multidrug-resistant *E. coli*.

## Introduction

Enteroaggregative *Escherichia coli* (EAEC) cause a range of gastrointestinal symptoms including acute or persistent, watery or mucoid diarrhoea, often accompanied by severe abdominal pain [1]. Studies have shown that EAEC make a significant contribution to the burden of diarrhoeal disease globally, and are a common cause of travellers’ diarrhoea in the UK [2]. Certain sequence types (STs), such as ST38, also cause extra-intestinal infections, including sepsis and urinary tract infections [3]. There are no known animal reservoirs and transmission is most likely person-to-person via the faecal oral route.

The implementation of whole genome sequencing (WGS) at Public Health England (PHE) has improved surveillance of antimicrobial resistance (AMR) of gastrointestinal pathogens [4]. Analysis of these surveillance data indicates that the incidence of AMR in strains of *E. coli* belonging to the EAEC pathotype is high in comparison with other diarrhoeagenic *E. coli* pathotypes [2]. In 2017, we identified a strain of EAEC serotype O51 : H30 ST38, isolated from a patient who developed persistent diarrhoea shortly before returning to the UK from Pakistan, that had 12 AMR genetic determinants including *bla*_OXA-181_, *bla*_CMY-16_, *bla*_CTX-M-15_, *bla*_TEM-1_, *bla*_OXA-1_, *aadA1b*, *aac(6′)-Ib-*cr, *gyrA*[83 : S-L];*parC*[80 : S-I;84 : E-V], *qnrS1*, *dfrA1*, *tetA*, *sul2* and *catA* [2]. As described previously, the isolate was confirmed phenotypically by antimicrobial susceptibility testing to be resistant to the third-generation cephalosporins, ciprofloxacin, streptomycin, trimethoprim, tetracycline, sulphonamide and chloramphenicol [2].

From the analysis of the short-read sequencing data, it was not possible to determine the whether the resistance determinants in this multidrug-resistant (MDR) strain of EAEC were plasmid-encoded or if they had been incorporated into the chromosome. The aim of this study was to use single-molecule, nanopore sequencing to explore the genomic environment of the resistance determinants in this MDR strain.

## Methods

### DNA extraction and nanopore sequencing

DNA was extracted using the Wizard Genomic DNA Purification kit (Promega). Library preparation was performed using the 1D Genomic DNA sequencing kit SQK-LSK108 (Oxford Nanopore Technologies) with the omission of DNA shearing and DNA repair steps to prevent further DNA fragmentation. Library preparation was initiated at the DNA end-prep step using NEB repair modules (New England Biolabs). All bead washing steps were performed using AMPure XP beads (Beckman Coulter). The final 80 µl prepared library was sequenced. Sequencing was performed on the MinION using a FLO-MIN-106 R9.4 Flow cell (Oxford Nanopore Technologies) using the MinKNOW software for the full 48 h run time with no alterations to any voltage scripts.

### Genome assembly and annotation

Nanopore raw FAST5 reads were basecalled using Albacore (Oxford Nanopore Technologies) v1.2.1., converted to FASTQ formats using Poretools v0.6.0 and assembled using Unicycler v0.4.2 combining the long-read sequencing data with short-read data produced by Illumina sequencing [5, 6]. The genome was interrogated against the previously described PHE AMR gene reference database [2] using blast n (alignment length 100 %, query coverage and identity at 80 %), and annotated using Prokka v1.12 [7]. Further annotation was performed manually using the information from Prokka and National Center for Biotechnology Information (NCBI) blastn for any unknown coding DNA sequences. When complete, regions of interest were viewed by DNA features viewer v0.1.3 (https://github.com/Edinburgh-Genome-Foundry/DnaFeaturesViewer). Illumina sequencing and detection of AMR determinants from the short-read data were performed as described previously [2].

Long- and short-read FASTQ sequences have been deposited in the NCBI Sequence Read Archive under the BioProject PRJNA315192 (SRP071789), Sample: SRS2937479, Experiment: SRX3676443, Accession numbers Nanopore FASTQ: SRR6702264 and Illumina FASTQ: SRR5470155. Assembly accession numbers: CP026723.1 (chromosome) and CP026724.1 to CP026728.1 (five individual plasmids)

## Results and Discussion

The genome of EAEC O51 : H30 ST38 assembled into a single chromosomal contig of 5 492 922 bp and five plasmids (Table 1). Located on the chromosome at positions 2 087 027–2 109 149 were genes encoding *pap* pili/fimbriae, characteristic of extra-intestinal *E. coli*. The largest plasmid was 129 627 bp (p266917_2_01) and belonged to replicon type IncFIB based on the *repA* sequence. This plasmid carried known EAEC virulence genes, including the universal regulator *aggR*, the dispersin-encoding *aap*, the aggregative transported *aat* and the *aggA* genes encoding fimbriae type I. The remaining four plasmids (p266917_2_02, p266917_2_03, p266917_2_04 and p266917_2_05) were smaller in size ranging from 33 288 to 97 124 bp (Table 1). These plasmids have been previously described in *E. coli* and *Klebsiella* species, and isolated in globally dispersed regions from clinical samples, food, animals and the environment (Table 1).

**Table 1.** Plasmids identified in EAEC O51 : H30 ST38

Plasmid name	Inc type	Replicon type	Size (bp)	Notes
p266917_2_01	IncFIB	*repFIB*	129 627	pAA encoding *aggR*
p266917_2_02	IncY	*repA1*	97 124	CP009168 – Clinical isolate (USA)
				CP015997 – Isolated from a chicken
				CP012494 – Isolated from food
				KU980950 – Korean clinical MDR isolate
p266917_2_03	IncI1	*repA2*	83 010	CP021208 – Clinical isolate
				CP023362/CP023370 – MDR isolates from veterinary sources
				KY964068 – Isolated from a pig
p266917_2_04	IncX3	*repB*	51 479	Harbours *bla*_OXA-181_ and *qnrS1*
p266917_2_05	IncX4	*repE*	33 288	CP016037 – Clinical isolate (Germany)
				JX981514 – Isolated from cattle
				KM580533 – Isolated from a pig

**Fig. 1.**
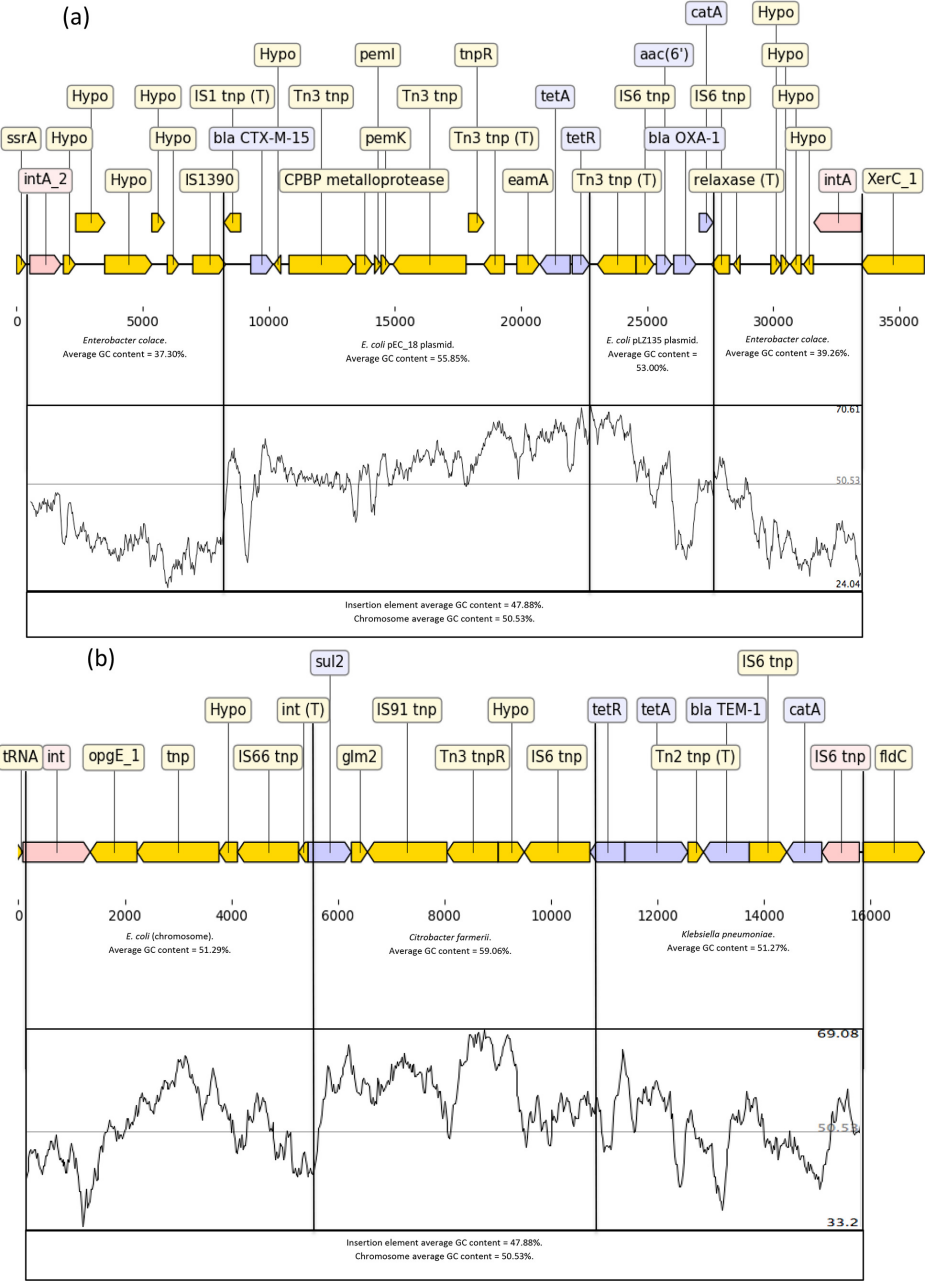
(a) Chromosomal AMR region 1 located at positions 1 686 326–1 719 287 between a transfer messenger RNA gene (*ssrA*) and a tyrosine recombinase (*xerC*) encoded *catA*, *bla*_OXA-1_, *aac(6′)-Ib-*cr, *tet*A and *bla*_CTX-M-15_. (b) Chromosomal AMR region 3 located at positions 3 674 464–3 693 662 between the tRNA-Leu (5′) gene and the (R)-phenyllactyl-CoA dehydratase beta subunit (*fldC*) (3′) gene encoded *catA*, *bla*_TEM-1_, *tet*A and *sul2* AMR determinants highlighted in blue and intergrases and insertion elements highlighted pink.

Despite the number of plasmids harboured by this isolate, analysis of the assembled genome indicated that the majority of the AMR determinants were clustered together on the chromosome in three separate regions flanked by integrases and/or insertion elements. AMR region 1 was located on the chromosome at positions 1 686 326–1 719 287 (32 961 bp) between a transfer messenger RNA gene (*ssrA*) and a tyrosine recombinase (*xerC*) (Fig. 1a). The AMR determinants located on this integron were *catA*, *bla*_OXA-1_, *aac(6′)-Ib-*cr, *tetA* and *bla*_CTX-M-15_, conferring reduced susceptibility to chloramphenicol, ampicillin, streptomycin and ciprofloxacin, tetracycline and the third-generation cephalosporins. *bla*_CTX-M-15_ is the most abundant CTX-M gene in ESBL (extended-spectrum beta-lactamase)-producing *E. coli* causing human infections [8]. Analysis using blastn indicated that the AMR determinants on this transposon originated from two different fragments of DNA originating from different plasmids (Accessions: NC_014384.1 and MF353155.1) inserted into a fragment of DNA that originated from a strain of *Enterobacter cloace* (Fig. 1a).

AMR region 2 was identified as a Class 2 Tn7 and encoded *dfrA1* and *aadA1*, conferring reduced susceptibility to trimethoprim and streptomycin. It was inserted at positions 3 044 997–3 057 993 (12 996 bp) between a phosphate-binding protein (*pstC*) and a glutamine-fructose-6-phosphate aminotransferase (*glmS*). Tn7 is a well-known representative of Class 2 integrons, and is found in many different bacterial species [9].

AMR region 3 was inserted between the tRNA-Leu gene and the phenyllactyl-CoA dehydratase beta subunit (*fldC*) gene at positions 3 674 464–3 693 662 (19 198 bp), and had four different AMR determinants, including *catA*, *bla*_TEM-1_, *tetA* and *sul2* conferring reduced susceptibility to chloramphenicol, ampicillin, tetracycline and sulphonamide (Fig. 1b). Analysis using blastn indicated that this region comprised DNA fragments exhibiting homology with DNA from *E. coli* and a fragment of plasmid DNA (Accession: CP015026.1) previously identified in *Klebsiella pneumoniae* and *Citrobacter farmerii* (Fig. 1b). It was observed that both *tetA* and *catA* were present at two separate locations on the chromosome (AMR regions 1 and 3) (Fig. 1a, b). Furthermore, there were multiple genes encoding reduced susceptibility to streptomycin [*aadA1b* and *aac(6′)-Ib-*cr], ciprofloxacin [*aac(6′)-Ib-*cr, *qnrS1* and the chromosomal mutations in *gyrA* and *parC*] and the β-lactams (*bla*_CMY-16_, *bla*_CTX-M-15_, *bla*_OXA-1_ and *bla*_TEM-1_) at different locations in the genome in this strain of EAEC (Fig. 1a, b).

The AMR determinants that were identified outside these three regions were *bla*_CMY-16_, located on the chromosome between positions 892 919 and 894 050, mutations in *gyrA* and *parC*, and two plasmid-encoded AMR determinants, the carbapenemase *bla*_OXA-181_ and the plasmid-mediated quinolone-resistant determinant (PMQR) *qnrS1* (Table 1). *bla*_CMY-16_ has been previously described in *Proteus mirabilis*, *Providencia stuartii*, *Salmonella enterica* and *Klebsiella pneumoniae* [10–12]. In *E. coli*, *bla*_CMY_ genes are usually found on plasmids but chromosomally encoded *bla*_CMY_ have been described [13].

In addition to the PMQRs *qnrS1* and *aac(6′)-Ib-*cr, which exhibit the potential to induce reduced susceptibility to the fluroquinolones, EAEC O51 : H30 also had one mutation in *gyrA*[83 : S-L] and two in *parC*[80 : S-I;84 : E-V]. The combination of all these fluroquinolone resistance determinants resulted in the isolate exhibiting an MIC>0.5 mg l^−1^ to ciprofloxacin.

Of the two plasmid-encoded AMR determinants, *bla*_OXA-181_ is a *bla*_OXA-48_-like carbapenemase conferring resistance to penicillins and carbapenems, and is commonly found on a 51 kb IncX3 plasmid [14], as observed in this study. The *bla*_OXA-181_ gene was initially identified in *Enterobacter cloacae* and *Klebsiella pneumoniae* isolates in India in 2007 [15]. *Enterobacteriaceae* isolates producing *bla*_OXA-181_ are globally distributed, although in most cases the patients report recent travel to the Indian subcontinent [16, 17]. The presence of *bla*_OXA-181_ on a self-transmissible IncX3 plasmid is of significance, as IncX3 plasmids have been found as a common vehicle mediating the dissemination of other carbapenemases, and have the potential to disseminate widely [15]. Due to the co-location of *qnrS1* on the same plasmid, treatment with ciprofloxacin may indirectly select for resistance to the carbapenems, as well as the fluroquinolones.

A previous study in the UK showed that *E. coli* ST38 was the ST most commonly associated with *bla*_OXA-48_-like genes, and 18 % (62/351) had *bla*_OXA-181_ [17]. The authors suggested that the accumulation of *bla*_OXA-48_-like carbapenemases within the UK is due to repeated importations, coupled with both the spread of successful clones (e.g. *E. coli* ST38) and, in particular, the dissemination of successful plasmids [17].

Finally, an assembly utilizing only the ONT reads was generated with Unicycler (v0.4.2) with default parameters. To estimate the accuracy of the derived assembly, Illumina reads were mapped using BWA v0.7.13 and Samtools v1.1 and a VCF file was generated using GATK v2.6.5 with true positive SNPs defined as those with a mapping depth greater than 10, a consensus variant ratio of greater than 0.9 and mapping quality greater than 30. Illumina reads could map accurately to 81.15 % of the assembly and 152 030 variants were identified. This corresponded to an error rate in consensus assembly sequence of 3.18 % [152 030/(5 880 795−1 108 320)×100=3.18].

### Conclusion

*E. coli* ST38 is a known cause of gastrointestinal disease and extra-intestinal infection, including sepsis and urinary tract infections, and has been associated with MDR in the UK and elsewhere [3, 18]. Short-read WGS data can identify the presence or absence of AMR determinants but not their genomic architecture. Long-read analysis of WGS data enables the characterization of mobile genetic elements on which the key AMR determinants are located, and identifies the combination of different AMR determinants co-located on the same mobile element, but may lack the accuracy required to identify AMR associated with mutations in chromosomal genes [19, 20]. A combination of short- and long-read WGS data facilitates the detailed analysis of AMR in *E. coli,* contributing to a better understanding of the transmission of co-located AMR determinants in MDR *E. coli* causing gastrointestinal and extra-intestinal infections.

## Data bibliography

1. Greig DR, Dallman TJ, Jenkins C. NCBI Sequence Read Archive PRJNA315192 (2018).
